# Rapid, multiplex and automated detection of bacteria and fungi in endophthalmitis via a microfluidic real-time pcr system

**DOI:** 10.1186/s12348-024-00446-6

**Published:** 2024-12-18

**Authors:** Siyu Wang, Yiteng Liu, Yingqi Li, Yibo Gao, Zhongliang Zou, Na Xu, Qi Song, Fangyan Liu, Yihong Song, Xian Wang, Zixin Fan

**Affiliations:** 1 Department of Ophthalmology, the First Affiliated Hospital of China Medical University, No. 155 Nanjing Bei Street, Heping District, Shenyang City, 110001 Liaoning Province PR China; 2 Division of Emerging Interdisciplinary Areas, Academy of Interdisciplinary Studies, The Hong Kong University of Science and Technology, Clear Water Bay, Kowloon, 999077 Hong Kong SAR China; 3 Thrust of Advanced Materials, The Hong Kong University of Science and Technology (Guangzhou), Nansha, Guangzhou, 511400 China; 4 The Affiliated Hospital of Guizhou Medical University, Guiyang, 550004 Guizhou Province China; 5 Department of Physics, The Hong Kong University of Science and Technology, Clear Water Bay, Kowloon, 999077 Hong Kong SAR China; 6 Ophthalmology Department, Shenzhen Eye Hospital, Shenzhen, 518040 Guangdong China; 7 HKUST Shenzhen-Hong Kong Collaborative Innovation Research Institute, Shenzhen, China

**Keywords:** Endophthalmitis, Microfluidics, Rapid detection, Lab-on-chip, Polymerase chain reaction

## Abstract

**Background:**

Endophthalmitis is an ophthalmologic emergency requiring accurate and rapid diagnosis for treatment. Currently, the diagnosis commonly relies on culture and molecular biology, which falls short of clinical rapid diagnosis. The purpose of this study was to evaluate the feasibility of a self-build Microfluidic Real-time Polymerase Chain Reaction (RT-PCR) System for rapidly identifying potential pathogens of endophthalmitis.

**Methods:**

This study included 22 patients who presented to Shenzhen Eye Hospital and the Ophthalmology Department of the Affiliated Hospital of Guizhou Medical University in China between January 2023 and March 2024. The samples were cultured using conventional methods and underwent Microfluidic RT-PCR and metagenomic next-generation sequencing (mNGS).

**Results:**

The Microfluidic RT-PCR System identified pathogens in 11 of 22 cases (50.00%), compared with 40.91% for microbiology culture. 14 cases (63.64%) had concordant results, and 5 cases were positive for the microfluidic system only. The agreements between culture and microfluidic system, as well as culture and mNGS were 100.00% (6/6) and 50.00% (3/6), respectively. The average waiting time for the microfluidic system was about 30 min if excepting DNA extraction time, which was much shorter than 2.88 days for culture and 1.57 days for mNGS.

**Conclusion:**

The microfluidic-based RT-PCR system was preliminarily proved to be a sensitive, easy-to-operate, and rapid in-hospital technology. It is expected to become a rapid diagnostic platform for endophthalmitis.

**Supplementary Information:**

The online version contains supplementary material available at 10.1186/s12348-024-00446-6.

## Introduction

Endophthalmitis is a medical emergency commonly associated with a bacterial or fungal infection of aqueous and/or vitreous humor [1]. It is characterized by blurred vision, red eyes, pain, photophobia, floaters, and swollen lids [2–5]. Endophthalmitis can be divided into endogenous and exogenous ones. Exogenous endophthalmitis is more common, which includes postoperative, traumatic, filtering bleb-associated, and corneal infection-related endophthalmitis [6, 7]. The incidence is highest after cataract surgery, especially when utilizing clear corneal incisions, and is lowest after pars plana vitrectomy (PPV) [8–10]. Endogenous endophthalmitis is a metastatic infection via the bloodstream. Gram-negative bacteria (like Klebsiella pneumoniae and Pseudomonas aeruginosa) and Gram-positive bacteria (like Staphylococcus epidermidis) are the leading causes in East Asia [11–14]. Accurately and rapidly detecting the pathogen is an essential condition for the treatment of endophthalmitis and plays a crucial role in subsequent therapies [15, 16]. Testing methods usually include sampling and culture techniques (aqueous and vitreous culture), as well as molecular biology, such as real-time polymerase chain reaction (RT-PCR) [17, 18].

Currently, most diagnoses of endophthalmitis rely on microscopy, culture, and sensitivity processes. Though culture is the gold standard for pathogen identification [19], it has several limitations. Firstly, a considerable portion of culture results are negative, which is about 63.5 to 77.5% for aqueous and 11.8 to 58.0% for vitreous samples, respectively [20–22]. These may be false-negative results or due to non-infectious inflammation, unknown pathogens, or prior antibiotic use. Meanwhile, culture is a process that takes 2 to 7 days, which is in contradiction with the urgent demand for timely treatment of such an emergency [23]. Next-generation sequencing (NGS) technology such as metagenomic next-generation sequencing (mNGS) [24, 25] enables efficient sequencing of large amounts of DNA to identify all potential pathogens in endophthalmitis samples, including bacteria, fungi, parasites, and viruses [26–28]. However, a high sensitivity and low specificity may also result in a high rate of false positives [29]. This application in endophthalmitis clinical diagnosis is also limited due to the complexity of the technology, high cost, and genetic ethical concerns [30, 31]. RT-PCR is another representative molecular biology examination which based on 16 S rRNA genes (bacteria) and 18 S.

rRNA genes (fungi and parasites). The process involves DNA extraction, sequencing, and matching against known pathogens in databases [32]. RT-PCR technology provides higher sensitivity than traditional culture methods and requires a smaller sample size. But it still falls short of clinical rapid diagnosis [33, 34]. Therefore, it is essential to explore new detection methods that are simple, rapid, cost-effective, and with high precision to assist clinical diagnosis and improve outcomes.

To overcome the drawbacks mentioned above, we introduced an improved microfluidic-based RT-PCR technology for rapid clinical diagnosis. Microfluidics is a technology that integrates traditional laboratory biochemical analysis processes onto a chip that is smaller than a few square centimeters or even [35]. By integrating microfluidic chip and quantitative RT-PCR technology, we could greatly simplify the process and achieve multiplex detection. These lab-on-a-chip applications are composed of micro/nano-channels or -grooves, in which fluids are driven by external force or micro-pumps/-valves for solutions mixing, separation, detection, etc. When performing RT-PCR, a flat double-layer design can offer a larger surface volume ratio and faster heat conduction, therefore reducing the amplification and detection time [36]. Additionally, the characteristics of small size and low material/sample consumption make it well-suited for multiplex PCR detection [37, 38]. However, conventional polymer materials have poor sealing performance, which may lead to evaporation and leakage since RT-PCR requires a temperature of 95 ℃. In this study, we used a silicon-glass chip to avoid this problem. Benefiting from miniaturization and rapidity, this technology can be completed within the hospital’s laboratory, significantly reducing waiting times. Moreover, due to the low cost of the chips, the testing is more cost-effective.

This study aimed to evaluate the feasibility of the Endophthalmitis Microfluidic RT-PCR System in rapidly identifying potential common pathogens of endophthalmitis, so as to improve the efficiency of diagnosis and guidance for treatment, especially for culture-negative cases. The results obtained from this system will also be compared with those from laboratory culture and mNGS.

## Materials and methods

This prospective, multicenter study included 22 patients with endophthalmitis who presented to Shenzhen Eye Hospital and the Ophthalmology Department of the Affiliated Hospital of Guizhou Medical University in China between January 2023 and March 2024. This study was approved by The Ethics Committee of Shenzhen Eye Hospital (2024KYPJ090) and conducted in adherence to the Declaration of Helsinki. All participants provided informed consent, and all diagnosis and treatment procedures were in accordance with clinical guidelines.

Inclusion criteria: (1) with endophthalmitis or suspected endophthalmitis; (2) accepted PPV due to endophthalmitis; (3) with written informed consent form.

Exclusion criteria: (1) exclusion of endophthalmitis diagnosis; (2) no need for PPV; (3) unstable overall condition; (4) refusal to participate in; (5) deemed unsuitable for the study.

### Data collection

The study collected demographic data, including gender, age, address, etc. Clinical information includes but is not limited to best-corrected visual acuity (BCVA), intraocular pressure (IOP), clinical manifestations, signs, underlying diseases, and predisposing factors (history of trauma, surgery, other systemic infections, etc.). All data were initially organized, processed, and stored by Excel software (Microsoft Corp., Redmond, WA, USA).

### Sample collection and storage

Depending on different patient conditions, intraocular fluid specimens were obtained through intraocular fluid biopsy (anterior chamber tap and/or vitreous tap), PPV, or a combination of both methods. For the intraocular fluid biopsy, a 1 ml syringe with a 30G needle was inserted into the anterior chamber or vitreous cavity to extract the specimens. For those who underwent PPV, specimens were obtained directly by vitrectomy probe or from the drain bag. During this process, the use of fluid/air exchange mode ensured the concentration of intraocular pathogens and avoided the impact of antibiotics in the infusion fluid. Specimens were stored at − 80 ℃ until use.

### Microbiology culture and metagenomic NGS

The intraocular fluid specimens were sent to the biological laboratory for routine microbiology culture processing. Firstly, the specimens were inoculated onto Columbia agar (for aerobic bacteria), CDC anaerobic blood agar (for anaerobic bacteria), and Sabouraud dextrose agar (for fungi) (Detgerm Microbiological Science Ltd., Guangzhou, Guangdong, China). Columbia agar and CDC anaerobic blood agar was incubated at 35 to 37 ℃ for 48 h, while Sabouraud dextrose agar was incubated at 28 to 31 ℃ for 7 days. All culture plates should be observed at least once a day. In cases of positive cultures, bacterial and/or fungal isolates were further identified using smear microscopic examination and VITEK-2 Compact system (bioMérieux, Marcy l’Etoile, France). Lactophenol cotton blue was used in the identification of filamentous fungi. According to clinical needs and with informed consent from the patients, intraocular specimens from a subset of patients (*n* = 7) were sent to an external testing laboratory (Giantmed Medical Diagnostics Lab, Beijing, China) for the mNGS. This sequencing technology utilizes NGS to compare nucleic acid sequences of pathogens with those in the databases, thereby identifying pathogens’ species [39, 40].

### Detection via microfluidic system

**Fig. 1:**
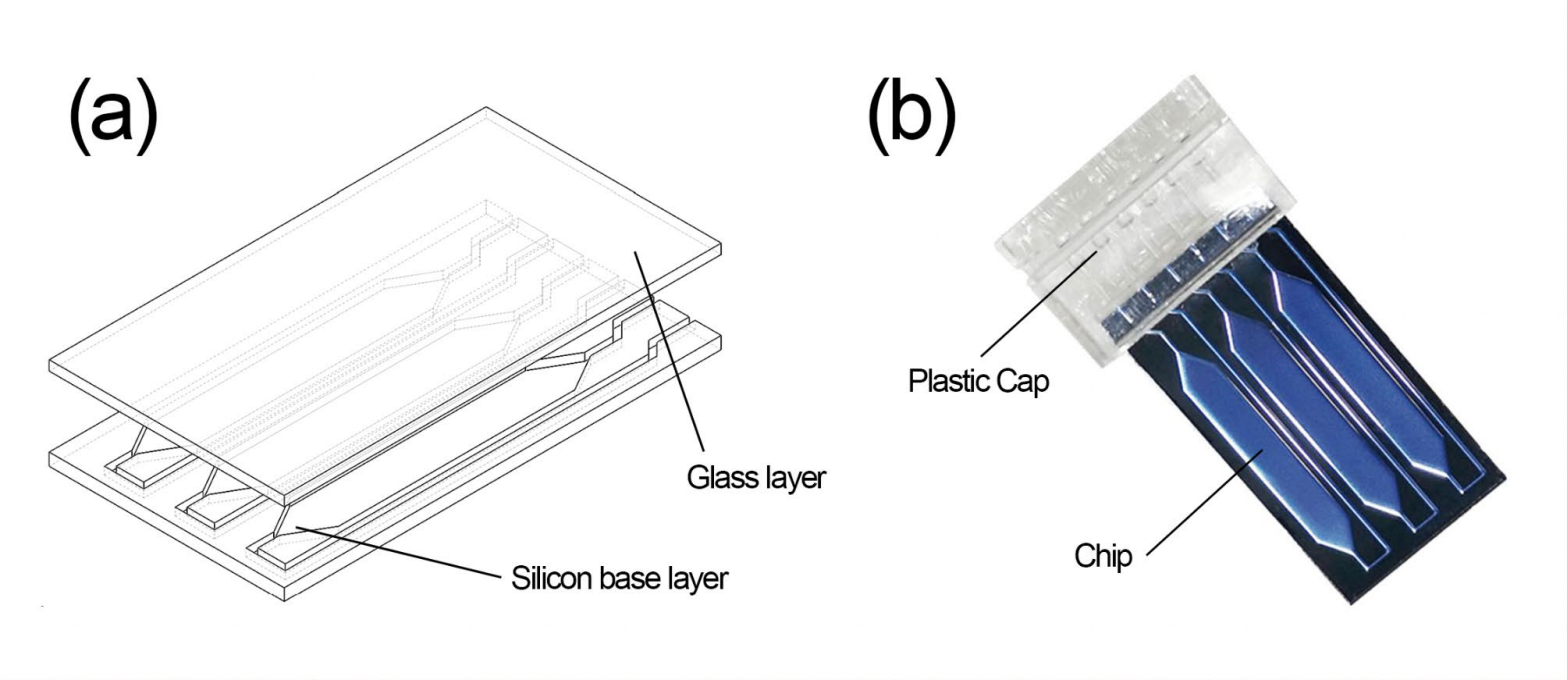
**(a)** Double-layer structure of the chip. The chip is composed of an upper layer made of glass and a lower layer constructed from silicon, with the reaction chamber and liquid flow channel integrated into the silicon substrate. **(b)** The photo of the microfluidic chip. The plastic cap consists of a joint and a cover, in the joint there are 6 channels connected to the 6 channels of the chip for liquid sampling and venting, and a cover for sealing the chip after sampling

**Fig. 2:**
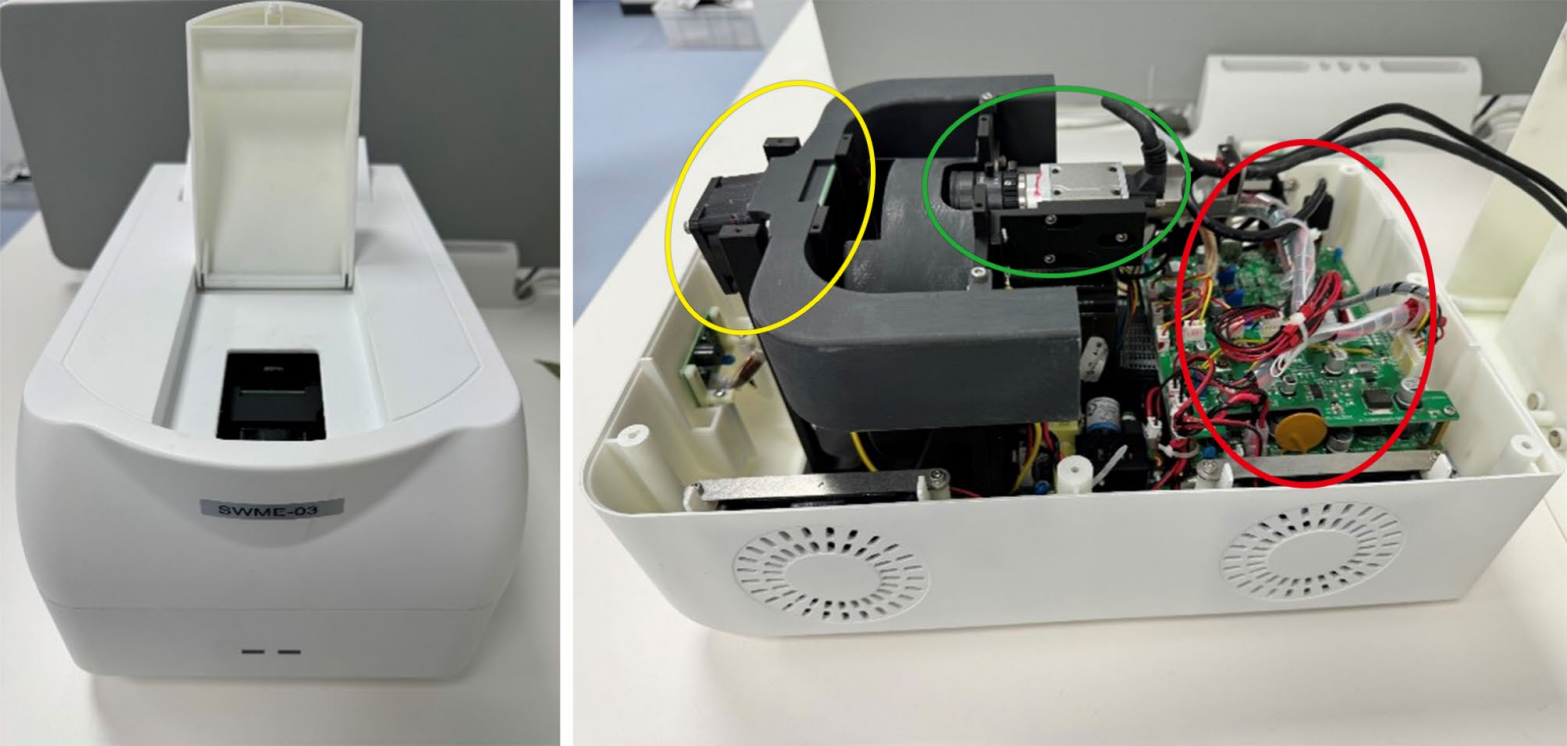
The overall structure of the microfluidic equipment: Heating module (yellow circle); Multicolor fluorescence detection module (green circle); Control board (red circle)

**Fig. 3:**
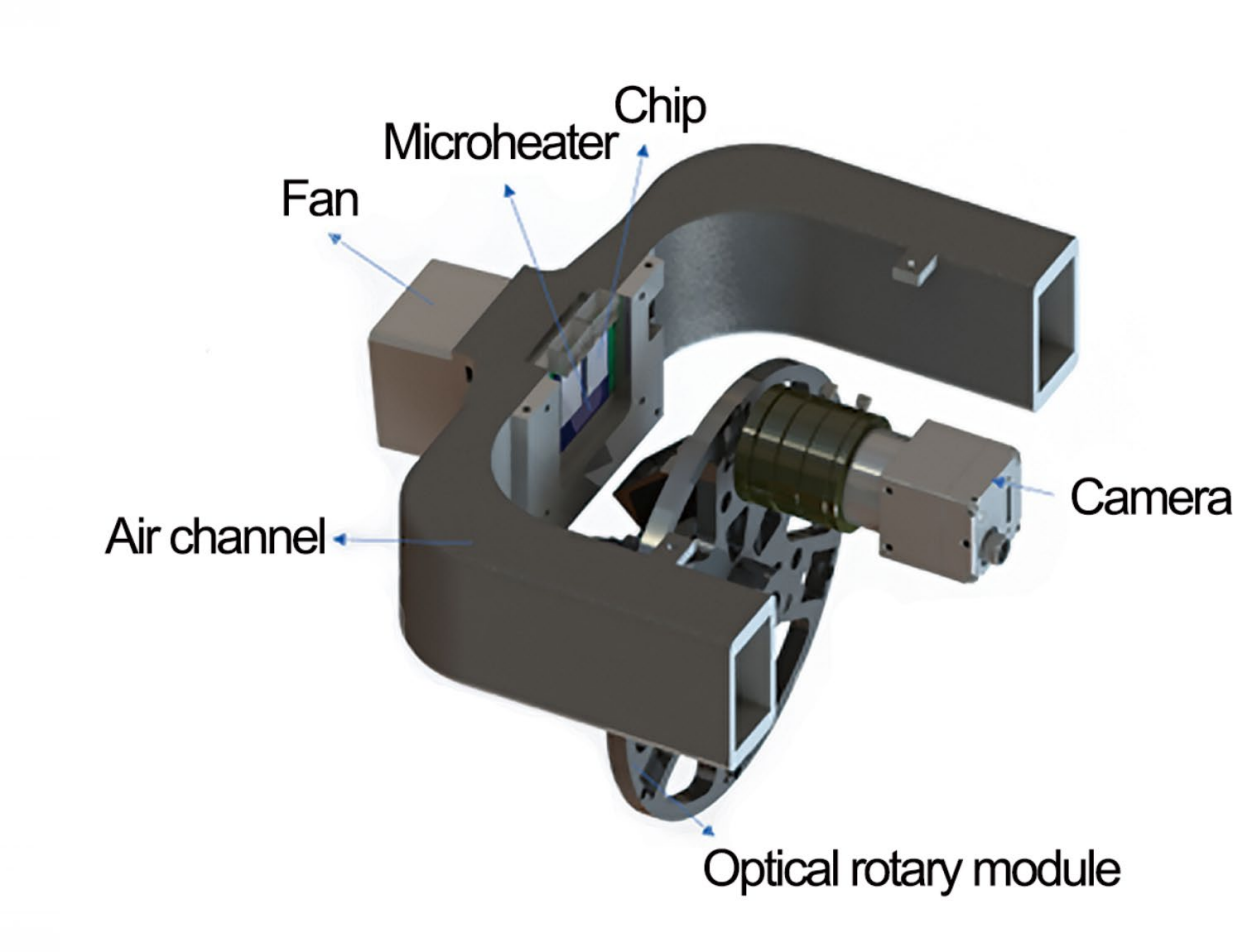
Structure of the heating module and multicolor fluorescence detection module. The heating module consists of a microheater and a fan, the air channel is used to quickly exhaust hot air during cooling. In the multicolor fluorescence detection module, an optical rotary module is fitted with different colored filters for multi-color fluorescence detection

The chips have a double-layer structure, with silicon base layers and glass layers, which are shown in Fig. 1 (a). The silicon layers are processed by photolithography and deep silicon etching technologies to develop microgrooves, and the reaction chambers are formed by bonding glass layers on the silicon layers after the surface oxidation process. Special plastic caps are designed for the addition of reagents and a fully enclosed reaction environment, which is shown in Fig. 1 (b) [41, 42]. The self-build microfluidic system is based on an equipment called SWME-03 (Shineway Technology Corp., Shenzhen, Guangdong, China) (Fig. 2) and a self-developed microfluidic chip (Fig. 1), utilizes a silicon-based platinum-patterned heater to achieve the functions of Joule heating and temperature sensing, which is manufactured by the lift-off process on the silicon substrate. A fan is utilized as a rapid cool-down device, and a proportional–integral–derivative controller is utilized to achieve precise temperature control [41, 43]. As shown in Fig. 3, SWME-03 utilizes a motor-driven rotary optical path module to achieve the detection of multicolor fluorescence intensity. A total of five kinds of fluorescence are capable of detecting, including 6-carboxyfluorescein (FAM), hexachlorofluorescein (HEX), carboxy-X-rhodamine (ROX), cyanine 5 (CY5), and cyanine 5.5 (CY5.5). In this research, FAM, HEX, and CY5 are used, which means that three sets of primer-probe are placed in one reaction chamber and a total of 18 targets (6 chambers ×3 fluorescence channels) could be tested in on go. Fluorescent images of the chip will be captured by the camera (IMX392 sensor, Sony Group Corporation, Tokyo, Japan), which will acquire an image at each fluorescent channel during every temperature cycle. The self-developed software will then process these images to extract real-time fluorescence intensity values from the reaction chamber, thereby enabling the determination of the cycle threshold (Ct) value for PCR.

The intraocular fluid specimen was first added to a nucleic acid extraction kit, and a 50 µL nucleic acid sample was obtained utilizing GenePure Pro fully automatic nucleic acid purification system (BIOER TECHNOLOGY, Hangzhou, Zhejiang, China). The sample is mixed with the reaction solution, enzyme mixture, template, and double-distilled water and then added into the chips. The reaction programs are then set up after the chip placed into the SWME-03, which included: (1) 1 cycle at 95 ℃ for 2–3 min; (2) 40 cycles at 95 ℃ for 5–10 s and 60 ℃ for 15–30 s, fluorescent intensity collection. We use 12.5 µL reagent with sample for one channel. The whole detection of 17 pathogens will use 6 channels (2 chips), and the total volume is 75 µL with 15 µL sample in it. There are two different reaction systems in the program, involving different reagent ratios and reaction times, which are presented together with the template and primer-probe sequences in Supplementary Table S1 to S3.

### Treatment and follow-up

Based on the patient’s medical history and clinical presentations, treatment plans were preliminarily selected, such as broad-spectrum antibiotic therapy. Treatment plans were then adjusted based on the results of microbiology culture and mNGS. The follow-up visits after treatment were at 1 week, 1 month, 3 months, and 6 months. BCVA, IOP, essential ophthalmic examination, and general condition were recorded at follow-up visits.

### Statistics and mathematical

IBM SPSS Statistics 25.0 software (IBM Corp., Armonk, NY, USA), STATA 15.0 software (Stata Corp., College Station, Texas, USA), Origin 2024 software (Origin Lab Corp., Northampton, USA) and GraphPad Prism 10 software (GraphPad Corp., San Diego, USA) were used for statistical analysis and graphical representation. Detailed descriptive statistics were performed. For the data of microbiology culture, and the microfluidic RT-PCR system, the McNemar-Bowker test was utilized to test the statistical difference, and Cohen’s kappa was used to test the agreement. For the data with mNGS results, Cochran’s Q test was utilized to test the statistical difference, and Fleiss’ Kappa to test the agreement. A correlation plot was made based on Kendall’s tau correlation analysis. *P* < 0.05 was considered statistically significant.

## Results

### ***Parameter of microfluidic system***

The microfluidic chip and temperature cycling system used in this article were demonstrated to have high reliability and be consistent with the LightCycler 480 (Roche, Basel, Switzerland) in the detection of COVID-19 (positive rates were 70.83% and 75%, respectively) [42]. In this research, a fluorescence-based multiplex microfluidic RT-PCR system was designed to detect 17 common pathogens of endophthalmitis (Staphylococcus epidermidis, Staphylococcus aureus, Candida albicans, genus Staphylococcus, Pseudomonas aeruginosa, genus Streptococcus, Streptococcus pneumoniae, Enterococcus faecalis, Stenotrophomonas maltophilia, Bacillus subtilis, Propionibacterium acnes, Klebsiella pneumoniae, genus Enterobacter, genus Mucor, genus Penicillium, genus Aspergillus, and genus Fusarium). The Preliminary experiment based on Staphylococcus aureus nucleic acid standard (BNCC371884, BeNa Culture Collection, Beijing, China) demonstrated a detection limit of about 500 copies/mL.

### Participants and samples

The study included specimens of intraocular fluid from 22 patients with endophthalmitis. The demographic data and clinical information of the patients are shown in Table 1. Among the 22 patients, 5 were female (22.73%) and 17 were male (77.27%). The mean age was 54.64 (range 22–84) years, with a median age of 55.50 years. The most common clinical history was eye trauma (59.09%), followed by post-surgery (22.73%). 21 patients (95.45%) had a baseline BCVA less than 20/200. One patient was diagnosed with endogenous endophthalmitis, which was caused by systemic infection after ureteral stone surgery. A total of 19 vitreous humor (86.36%) and 3 aqueous humor samples (13.64%) were collected and aliquoted for different detections.

**Table 1:** Demographics and Clinical Data of the Endophthalmitis patients

ID	Age	Gender	Diagnosis	Laterality	Clinical history	Duration	Initial BCVA	Main treatment	Final BCVA
**1**	84	M	Exogenous endophthalmitis	OS	NA	1 day	LP	PPV	LP
**2**	59	M	Exogenous endophthalmitis; IOFB	OD	Trauma	2 days	HM	PPV	FC
**3**	34	M	Exogenous endophthalmitis	OD	Trauma	10 days	HM	PPV + IVC + IVV	HM
**4**	56	M	Exogenous endophthalmitis; IOFB	OD	Trauma: wood	1 week	LP	PPV	HM
**5**	60	M	Exogenous endophthalmitis; IOFB	OS	Trauma: iron nail	16 days	HM	PPV + IVC + IVV	HM
**6**	72	M	Exogenous endophthalmitis;	OD	Post PEA and IOL implantation	3 days	LP	PPV + IVC + IVV V ivgtt	FC
**7**	45	M	Exogenous endophthalmitis	OD	NA	2 days	HM	PPV + IVC + IVV C ivgtt	20/160
**8**	22	M	Exogenous endophthalmitis	OS	Post PC-IOL implantation	3 days	HM	PPV + IVC + IVV C ivgtt + V ivgtt	20/200
**9**	80	F	Uveitis	OD	NA	6 years	20/2000	Diagnostic PPV	20/250
**10**	36	M	Exogenous endophthalmitis; IOFB	OS	Trauma: aluminum can	2 days	HM	PPV + IVV L ivgtt + D ivgtt	20/100
**11**	45	M	Exogenous endophthalmitis	OS	Trauma: steel wire	1 days	LP	PPV + IVC + IVV C ivgtt + V ivgtt	NLP
**12**	68	M	Exogenous endophthalmitis	OD	Post corneal transplantation	15 days	HM	Vor ivgtt	20/500
**13**	71	F	Exogenous endophthalmitis	OD	Post PEA and IOL implantation	8 days	20/1000	PPV + IVC + IVV C ivgtt + V ivgtt	20/60
**14**	73	F	Endogenous endophthalmitis;	OU	Systemic infection after ureteral stones surgery	10 days	20/100	C ivgtt	OD 20/32 OS 20/50
**15**	42	M	Exogenous endophthalmitis	OD	Trauma: steel wire	2 days	FC	IVC + IVV	20/2000
**16**	35	M	Exogenous endophthalmitis	OD	Trauma: steel wire	16 days	20/400	PPV + IVC + IVV C ivgtt + Vor ivgtt	20/1000
**17**	50	M	Exogenous endophthalmitis; IOFB	OS	Trauma: metal fragment	5 days	HM	PPV + IVC + IVV	20/32
**18**	60	M	Exogenous endophthalmitis; IOFB	OS	Trauma	10 h	HM	PPV + IVC + IVV	20/100
**19**	51	M	Exogenous endophthalmitis	OD	Trauma: iron nail	13 days	LP	PPV + IVC + IVV CRO ivgtt + D ivgtt Itr PO	Evisceration
**20**	55	F	Exogenous endophthalmitis	OS	Post corneal laceration repair	2 weeks	HM	PPV + IVC + IVV C ivgtt + V ivgtt	HM
**21**	77	F	Exogenous endophthalmitis	OS	Trauma: chestnut	1 day	LP	PPV + IVC + IVV Vor ivgtt	FC
**22**	27	M	Exogenous endophthalmitis	OD	Trauma: iron fragment	2 days	HM	PPV + IVC + IVV L ivgtt	HM

M: male; F: female; IOFB: intraocular foreign body; OS: oculus sinister; OD: oculus dextrum; NA: not applicable (missing details); PEA: phacoemulsification and aspiration; IOL: intraocular lens; BCVA: best-corrected visual acuity; HM: hand movements; LP: light perception; NLP: no light perception; PPV: pars plana vitrectomy; IV: intravitreal injection; C: ceftazidime; V: Vancomycin; L: levofloxacin; CRO: ceftriaxone; vor: voriconazole; D: dexamethasone; Itr: itraconazole; ivgtt: intravenously guttae; PO: per os

### Basic results of the microfluidic system, culture and mNGS

**Fig. 4:**
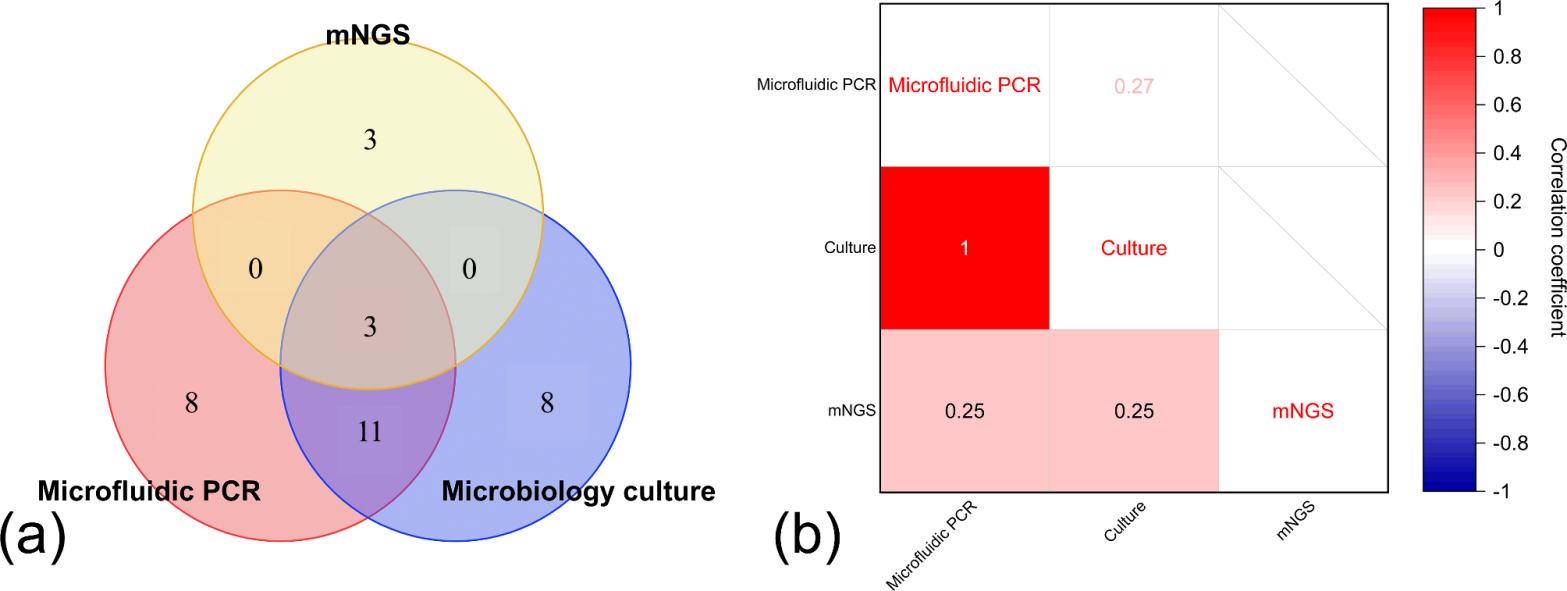
**(a)** Venn diagram among microbiology culture, mNGS and microfluidic RT-PCR: The frequency of consistent results among the three methods. **(b)** Correlation plot for the agreement among microbiology culture, mNGS and microfluidic RT-PCR: The lower left represents the correlation of 6 samples that underwent all three exams, with a correlation coefficient of 1.00 (*P* < 0.05) between culture and microfluidic RT-PCR, and 0.25 (*P* = 0.62 > 0.05) between mNGS and the other two exams. The upper right shows the correlation coefficient between microfluidic RT-PCR and culture for all 22 samples, with a result of 0.27 (*P* = 0.21 > 0.05), and mNGS were not included in this analysis

A total of 21 out of 22 received microbiology culture in our centers. One patient only received mNGS detection because the culture had been completed in other hospitals, and resulted negative. Nine specimens were culture-positive (40.91%), among which the most common pathogen was Staphylococcus epidermidis (4/9, 44.44%), and others included Proteus mirabilis, Aspergillus fumigatus, Aspergillus flavus, Acinetobacter lwoffii, and Streptococcus salivarius. The waiting time for microbiology culture was 2.88 (range 2 to 4) days. For the microfluidic system, 11 out of 22 (50.00%) specimens resulted positive, with the most common also being Staphylococcus epidermidis (4/11, 36.36%), and the others included genus Aspergillus, genus Mucor, genus Streptococcus, Enterococcus faecalis, genus Staphylococcus, and Propionibacterium acnes. The average waiting time was less than 1 h and only about 30 min if excepting DNA extraction time. Six out of 22 (27.27%) of the patients agreed and accepted the mNGS examination, of which only 1 specimen was positive for microbiology culture. Four out of 6 specimens (66.67%) tested positive in mNGS, identifying Streptococcus salivarius, Human betaherpesvirus 5 along with Human gammaherpesvirus 4 (Epstein–Barr virus), Candida albicans, and Moraxella nonliquefaciens. The average waiting time for mNGS was 1.67 (range 1 to 3) days. All results are shown in Table 2; Fig. 4 (a).

**Table 2:** Results of microbiology culture, mNGS and microfluidic RT-PCR system

ID	Sample	Microfluidic RT-PCR	Microbiology culture	mNGS
**1**	AH	genus Staphylococcus; genus Enterobacter	Negative	NA
**2**	VH	Staphylococcus epidermidis	Staphylococcus epidermidis	NA
**3**	VH	genus Streptococcus	Negative	NA
**4**	VH	Staphylococcus epidermidis	Staphylococcus epidermidis	NA
**5**	AH	Negative	Negative	Negative
**6**	VH	genus Streptococcus	Streptococcus salivarius	Streptococcus salivarius
**7**	VH	Negative	Negative	Moraxella nonliquefaciens
**8**	VH	Negative	Negative	Negative
**9**	VH	Negative	Negative	Human betaherpesvirus 5; Human gammaherpesvirus 4
**10**	VH	Propionibacterium acnes	Negative	NA
**11**	VH	Negative	Proteus mirabilis	NA
**12**	AH	Negative	Aspergillus fumigatus	NA
**13**	VH	genus Aspergillus	Negative	NA
**14**	VH	Negative	Negative	Candida albicans
**15**	VH	Negative	Acinetobacter lwoffii	NA
**16**	VH	Staphylococcus epidermidis	Staphylococcus epidermidis	NA
**17**	VH	Negative	Negative	NA
**18**	VH	Staphylococcus epidermidis; genus Staphylococcus	Staphylococcus epidermidis	NA
**19**	VH	genus Aspergillus	Aspergillus flavus	NA
**20**	VH	Negative	Negative	NA
**21**	VH	genus Mucor	Negative	NA
**22**	VH	Negative	Negative	NA

AH: aqueous humor

VH: vitreous humor

NA: patients did not accept the examination

mNGS: metagenomic next generation sequencing

### Agreement between microbiology culture and microfluidic system

The positive rates of the microfluidic system and microbiology culture were 50.00% (11/22) and 40.91% (9/22), respectively. The most common results of both examinations were Staphylococcus epidermidis. Of the 22 patients who received both microbiology culture and microfluidic exams, 14 patients (63.64%) showed concordant results between these two examinations, of which 6 were positive and 8 were negative. Three specimens were positive for microbiology culture but negative for microfluidic system (Proteus mirabilis, Acinetobacter lwoffii, and Aspergillus fumigatus). In contrast, five specimens were negative for microbiology culture but positive for microfluidic system (genus Aspergillus, genus Mucor, genus Streptococcus, Propionibacterium acnes, and Enterococcus faecalis) (Tables 2 and 3). The mcNemar-Bowker test could not reveal a statistical difference between the two methods (*P* = 0.73 > 0.05). Cohen’s kappa test results did not show statistically significant agreement between the two methods (*P* = 0.19 > 0.05), and the kappa value was 0.27.

**Table 3:** Comparison of microbiology culture, mNGS and microfluidic RT-PCR results

		Microbiology culture			mNGS				
		Positive	Negative	Total		Positive	Negative	Total	
**Microfluidic RT-PCR**	**Positive**	6	5	11		1	0	1	
	**Negative**	3	8	11		3	2	5	
	**Total**	9	13	22		4	2	6	

mNGS: metagenomic next generation sequencing

RT-PCR: real time-polymerase chain reaction

### Agreement between microbiology culture, mNGS, and microfluidic system

For the 6 specimens that underwent culture, microfluidic, and mNGS testing (Case 5, 6, 7, 8, 9, and 14), only 1 specimen showed positive results in all three tests, which was Streptococcus salivarius. Two specimen showed negative results in three tests, and only mNGS tests were positive for the remaining 3 specimens (Table 2). The consistency rates of microbiology culture with mNGS and microfluidic RT-PCR results were 50.00% (3/6) and 100.00% (6/6), respectively. Cochran’s Q test could not prove that there was no statistically significant difference among the three methods (*P* = 0.049 < 0.05). Fleiss’ Kappa test indicated no statistically significant agreement among the three methods (*P* = 0.29 > 0.05), and the kappa value was 0.25. The correlation coefficient of culture and microfluidic RT-PCR was 1.0 (*P* < 0.05), while correlation coefficient of culture and mNGS, as well as microfluidic RT-PCR and mNGS was 0.25 (*P* = 0.62 > 0.05), which were shown in Fig. 4 (b).

### Treatment and prognosis

**Fig. 5:**
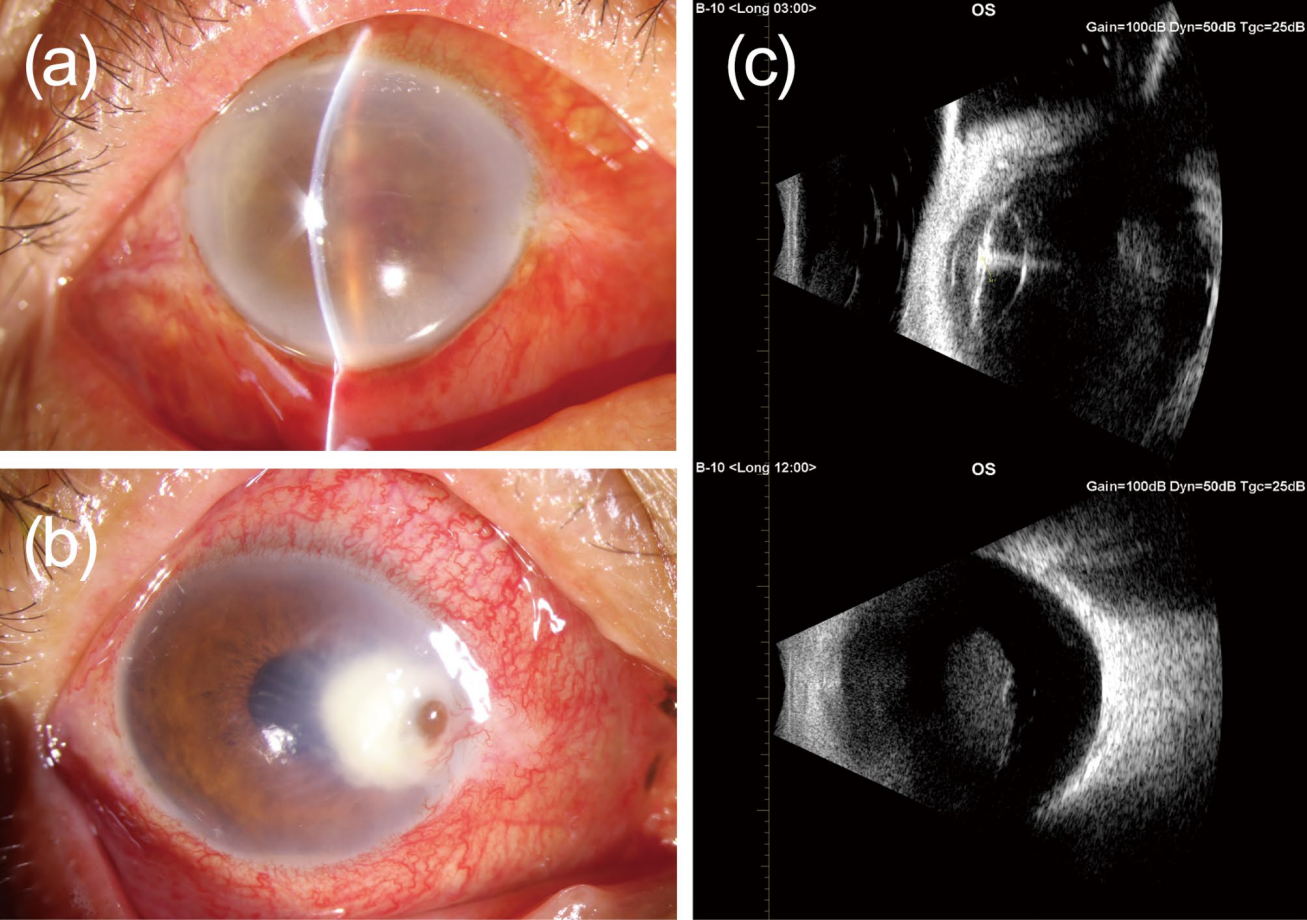
**(a)** Anterior segment photography of case 6: Conjunctival injection, corneal edema, aqueous flare, hypopyon about 1 mm, and exudation obscuring the pupil is observed in the right eye; **(b)** Anterior segment photography of case 12: Conjunctival injection, ulcerative lesion about 5 × 5 mm at nasal limbus, corneal perforation with iris prolapse, neovascularization, aqueous flare, hypopyon, and posterior synechia is observed in the right eye; **(c)** B-scan ultrasonography of case 10: Vitreous opacities in the left eye

Before presenting to our center, one patient received systemic anti-infection management (case 12, antifungal treatment). For patients suspected or diagnosed with endophthalmitis, 19 (86.36%) were treated with PPV, 16 (72.72%) received intravitreal injections (vancomycin 1.0 mg/0.1 ml and/or ceftazidime 2.2 mg/0.1 ml) and 15 (68.18%) underwent both treatments simultaneously. For two patients suspected of fungal endophthalmitis, voriconazole and itraconazole were added for treatment. For patients diagnosed with endogenous endophthalmitis, intravenous ceftazidime (1.0 g q8h) was administered. One patient was diagnosed with uveitis and accepted diagnostic PPV. Topical antibiotic eye drops treatments included antibiotic eye drops (Ofloxacin, etc.), steroid eye drops (Tobradex, etc.), and so on. The examination details of cases 6, 10, and 12 are shown in Fig. 5. Of the 22 patients with complete follow-up information, 15 patients improved their vision acuity, and 5 patients did not have improvement. One patient lost light perception but could preserve the eyeball, and one patient had to undergo evisceration. More details are shown in Table 1.

## Discussion

Endophthalmitis is an extremely serious and urgent ophthalmic emergency, which can lead to irreversible vision loss if not treated promptly. It can be divided into endogenous and exogenous endophthalmitis [1, 44]. Currently, the diagnosis of endophthalmitis mainly depends on the patient’s history, clinical manifestations and signs, and microbiology culture [45, 46]. Once suspected or diagnosed with endophthalmitis, in addition to timely treatment, the detection of pathogens is a crucial step in developing or changing the treatment plan, and influencing the prognosis [11, 47]. The testing methods currently mainly consist of microbiology culture and molecular biology techniques [17, 48].

Microbiology culture, as the gold standard for diagnosing endophthalmitis, has several disadvantages, such as long culture waiting time and high negative diagnostic rate. During the waiting period for culture results, there is a high likelihood of delaying treatment or changes in the disease condition so that the original culture results are no longer applicable [26, 49, 50]. Molecular biology techniques also play a significant role, especially in the diagnosis of culture-negative cases. Since the first application of PCR in the detection of pathogens in ophthalmology in 1991 [51], it has successfully increased the positive rate of diagnosis of endophthalmitis and shortened the time [33, 34, 52]. NGS technology, particularly mNGS, can simultaneously detect a variety of microorganisms in 1 specimen. It not only identifies pathogens but also microorganisms unrelated to endophthalmitis and drug-resistance genes. However, its drawback lies in its high technical requirements, making it challenging to implement in the hospital [53–55].

Microfluidic-based RT-PCR system, compared to the technologies mentioned above, has the advantages of cost-effectiveness, miniaturization, automation, multiplex, and simple operation. Compared to the testing methods in our study (an average costs of $ 26.32 for culture and $ 490.00 for mNGS), our microfluidic system enables a cost about $ 6.00, thanks to the mature semiconductor processing technology. On the basis of traditional quantitative RT-PCR, microfluidic-based RT-PCR further enhanced the advantages and improved shortcomings such as long detection time and complicated procedures, making it possible to perform rapid preliminary pathogen detection inside hospitals. By improving the chip structure, heating devices, and optical path, the difficulty of operation and detection efficiency can be further enhanced [41–43]. Fluorescence-based PCR detection allows for highly sensitive and specific quantitative detection. Due to the limited number of fluorescent probes and the need for different primers, the number of pathogens that can be simultaneously detected is limited. However, this does not affect its role as a novel method for rapid preliminary diagnosis. The comparison of microbiology culture, mNGS, traditional quantitative RT-PCR and microfluidic RT-PCR system is shown in Table 4.

**Table 4:** Comparison of microbiology culture, metagenomic NGS, traditional qRT-PCR and microfluidic RT-PCR system

	Microbiology culture	Metagenomic NGS	Traditional qRT-PCR	Microfluidic RT-PCR
**Technique**	By observing the growth of pathogens in the agar	By comparing nucleic acid sequences of pathogens with those in the databases	By detecting whether a specific fluorescent probe exceeds a threshold during RT-PCT	By simultaneously detecting multiple specific fluorescent probes via microfluidic chips
**Advantage**	Traditional gold standard; able to test antibiotic; cost-effective	Sensitive; wide pathogen spectrum; able to test antibiotic; susceptibility	Sensitive; quantitative; relatively fast	Sensitive; short time consuming; multiplex; easy operation; wider pathogen spectrum
**Limitation**	High false negative rate; long time consuming; susceptibility	High cost; require professional facilities; ethical problem	Limit pathogen spectrum; unable to test antibiotic; susceptibility	Unable to test antibiotic; susceptibility
**Speed**	Days to weeks	Days	Hours	Less than 1 h
**Accessibility**	Inside hospital	Specific centers	Inside hospital	Inside hospital/operating room/bedside

NGS: next generation sequencing

RT-PCR: real time-polymerase chain reaction

qRT-PCR: quantitative real time-polymerase chain reaction

In this study, the diagnostic positive rate of the microfluidic system (50.00%) was higher than that of the microbiology culture (40.91%). The results of 14/22 specimens were consistent, and the pathogen detection of both samples was consistent when both detections were positive. Staphylococcus epidermidis was the most common pathogen in this study, which is consistent with other reports [56]. That confirmed the accuracy and sensitivity of the microfluidic system, but we have not been able to statistically confirm it. Three specimens were positive for microbiology culture and negative for the microfluidic RT-PCR system, two of which were due to the detection of pathogens being beyond the detection spectrum of the microfluidic system. And the other one was suspected to be due to: (1) Insufficient sample volume, only about 20 µL for the poor cooperation; (2) Receiving empirical antifungal treatment before sampling. Five specimens were positive for the microfluidic RT-PCR system but negative in culture. We consider that might be due to the false negatives of the culture. Taking cases 10 and 21 as examples, Propionibacterium acnes and genus Mucor were detected in these two cases by microfluidic RT-PCR respectively, and received corresponding antibacterial and antifungal treatments. Both cases experienced improved BCVA postoperatively, indirectly confirming a higher positive rate and accuracy of this system. The overall waiting detection time of the microfluidic system is 0.5–1 h, which is much shorter than the average time for microbiology culture. These results suggest that microfluidic systems can provide helpful information on the detection of the pathogens associated with endophthalmitis and have a higher sensitivity and shorter detection time than microbiology cultures.

In two cases, more than one pathogen was detected via the microfluidic system, while some of these pathogens had not been found during microbiology culture (Cases 1 and 18). This might be because of different growth rates and competition among pathogens or human negligence. As long as the pathogen is within the spectrum, the microfluidic system can successfully detect more than one pathogen simultaneously, which had not been achieved via microbiology culture in our study.

The positive rate of mNGS was the highest among these three methods. Results were consistent for 3 out of 6 specimens, with only 1 specimen showing positive results in all three testing methods. The mNGS successfully detected virus specimens and provided results of suspected commensal microorganisms in all 6 specimens. Although mNGS has a high sensitivity and is able to detect viruses, there may be a possibility of false positivity [29, 57]. As in this study, based on the gold standard microbiology culture, the consistency rate of microfluidics RT-PCR was higher than that of mNGS. This could be due to the bias of a small sample size, but it is also not to rule out the situation of false positives. These results show that the microfluidic system is not comparable to mNGS detection in terms of sensitivity and spectrum range, but its advantages lie in a more concise process and shorter detection time.

This study has several limitations. Firstly, the sample size was relatively small. Secondly, for economic reason or patients’ demand, only limited samples underwent three examinations simultaneously. Thirdly, we had not conducted direct microscopy tests, and for endogenous endophthalmitis, a systematic blood culture was not performed. Larger samples and longer follow-up times are needed to address the mentioned issues and further confirm our results.

In conclusion, this study confirms the practicality of the microfluidic-based RT-PCR system in the identification of pathogens in intraocular fluid of suspected endophthalmitis. The higher sensitivity and faster detection speed make it not only a supplementary detection method for microbiology culture but even superior in some conditions especially when emergency. Compared with mNGS, the relatively concise process makes it possible to complete the test in the hospital. Therefore, it holds significant clinical importance for early diagnosis and appropriate treatment of endophthalmitis. With the expansion of the pathogen spectrum in the future, its testing range and sensitivity can be further enhanced.

## Electronic supplementary material

Below is the link to the electronic supplementary material.

Supplementary Material 1

## Data Availability

The datasets used and analysed during the current study are available from the corresponding author on reasonable request.

## Abbreviations

PCR: Polymerase chain reaction
PPV: Pars plana vitrectomy
RT-PCR: Real-time-polymerase chain reaction
NGS: Next-generation sequencing
mNGS: metagenomic next-generation sequencing
BCVA: Best corrected visual acuity
IOP: Intraocular pressure
FAM: 6-carboxyfluorescein
HEX: Hexachlorofluorescein
ROX: carboxy-X-rhodamine
CY: Cyanine
Ct: Cycle threshold
