# Supplementary material for: Rapid, multiplex and automated detection of bacteria and fungi in endophthalmitis via a microfluidic real-time pcr system
Source: J Ophthalmic Inflamm Infect. 2024 Dec 18;14:64. doi: 10.1186/s12348-024-00446-6 (PMC11655742; doi:10.1186/s12348-024-00446-6)
Supplement: Supplementary file 1 — Supplementary Material 1 [file 12348_2024_446_MOESM1_ESM.docx]

| **ID** | **Pathogen** | **Fluorescence** | **Primer name** | **Sequence** | **Probe** |
| --- | --- | --- | --- | --- | --- |
| **1** | genus Streptococcus | CY5 | Strep F | AGAAAGGGACGGCTAACTA |  |
|  |  |  | Strep R | CTCSCTTTACGCCCAATA |  |
|  |  |  | Strep PbR | CGGACAACGCTCGGGACCTACG | 5’CY5;3'BHQ2 |
| **2** | Staphylococcus aureus | HEX | Sau Pb | ACAGTGCAACTTCAACTAAAAAATTAC | 5' 6-HEX;3'BHQ1 |
|  |  |  | Sau F | AAGTGRTTCTGAAGATCCAACA |  |
|  |  |  | Sau R | TAATGTCGCAGGTTCTTTATGT |  |
| **3** | Staphylococcus epidermidis | FAM | SE Pb | ACAGATTCTATTACGCCACAACAACTCAT | 5’6-FAM;3'BHQ1 |
|  |  |  | SE F | CGTGAAAGAATGTCAATACAAGA |  |
|  |  |  | SE R | GATTGATGCAATAACTGGTCTG |  |
| **4** | Pseudomonas aeruginosa | HEX | PA Pb | TGTTCCACCGCRGTCTTCACCTCG | 5' 6-HEX;3'BHQ1 |
|  |  |  | PA F | CTCGCAGACCAAGGACAA |  |
|  |  |  | PA R | CAGGAAGTCGGCGAAGTA |  |
| **5** | Candida  albicans | CY5 | CA Pb | TTGTCCCTTAGTGTTACACAACAGA | 5’CY5;3'BHQ2 |
|  |  |  | CA F | GAAACTGTCATTGATGGCATTA |  |
|  |  |  | CA R | TGTAAACAAATGGAACCCCT |  |
| **6** | Genus  Aspergillus | FAM | Aspergillus Pb | TCATCGAGTCTTTGAACGCACATTG | 5’6-FAM;3'BHQ1 |
|  |  |  | Aspergillus F | TGAAGAACGCAGCGAAATG |  |
|  |  |  | Aspergillus R | CTTGAGGGCAGCAATGAC |  |
| **7** | Genus  Staphylococcus | FAM | Staphy PbR | CGCCTTCGCCACTGGTGTTCC | 5’6-FAM;3'BHQ1 |
|  |  |  | Staphy F | CGTGGAGGGTCATTGGAAA |  |
|  |  |  | Staphy R | ACATCAGCGTCAGTTRCAGA |  |
| **8** | Streptococcus pneumoniae | FAM | Spn PbR | CCAACAAATCGTTTACCGCTCCGC | 5’6-FAM;3'BHQ1 |
|  |  |  | Spn F | CTGGTTTGGCAAGTAGYGATAGC |  |
|  |  |  | Spn R | CTGGGACATTATTGACCTGACCATA |  |
| **9** | Enterococcus faecalis | HEX | Efs-Pb | TTCCGTTGGTCTGGCGTCCCTTTCT | 5' 6-HEX;3'BHQ1 |
|  |  |  | Efs-F | CACTGAAACTTTTGTTGCTGGTAAA |  |
|  |  |  | Efs-R | CGATGTTAATRCGTGTGCCTT |  |
| **10** | Stenotrophomonas  maltophilia | CY5 | Sm-PbR | CAGCACAACCTCGGCAGATTCGC | 5’CY5;3'BHQ2 |
|  |  |  | Sm-F | GCATCTACATCGAGATCAAGCG |  |
|  |  |  | Sm-R | GATTCCATCTGCGTCTGCTG |  |
| **11** | Bacillus  subtilis | FAM | Bs Pb | TGATTACGATCTGCTTGCCAACCGC | 5’6-FAM;3'BHQ1 |
|  |  |  | Bs Pb b | TGATTATGATCTGCTTGCBAACCGC | 5’6-FAM;3'BHQ1 |
|  |  |  | Bs F | CCCTGAAATTTTCTCAGAAACAAC |  |
|  |  |  | Bs R | CTTGTCCTTCACGTTTATCTTCA |  |
| **12** | Propionibacterium acnes | HEX | P.acnes Pb | CCACACGCCTACCACGATACGACAC | 5' 6-HEX;3'BHQ1 |
|  |  |  | P.acnes F | GCCACAACAACCTAGAGTCT |  |
|  |  |  | P.acnes R | CGAACGGAATTAAGTCTGTAAGTC |  |
| **13** | Klebsiella pneumoniae | CY5 | Kp-Pb | ACCGTTGATCCGCTCCGTGCTG | 5’CY5;3'BHQ2 |
|  |  |  | Kp-F | GCARTGGATGGTGGACGAT |  |
|  |  |  | Kp-R | GGTCTTATCGGCGATAAACCAG |  |
| **14** | Genus Enterobacterium | HEX | Ent Pb | TGAGAGGATGACCAGCCACACT | 5' 6-HEX;3'BHQ1 |
|  |  |  | Ent F | ATCAGATGTGCCCAGATGG |  |
|  |  |  | Ent R | TGGACCGTGTCTCAGTTC |  |
| **15** | Genus  Mucor | FAM | Mucor PbR | CCAAGAATTTCACCTCTAGCGACCA | 5’6-FAM;3'BHQ1 |
|  |  |  | Mucor F | AGTAATGATGAATAGAAACGGTTGG |  |
|  |  |  | Mucor R | GCTTTCGCAGTAGTTTGTCTT |  |
| **16** | Genus  Penicillium | CY5 | Penicil PbR | TGCCGCCGCTGCCTTTCG | 5’CY5;3'BHQ2 |
|  |  |  | Penicil F | GTCATTGCTGCCCTCMAG |  |
|  |  |  | Penicil R | GTGACRAAGCCCCATACG |  |
| **17** | Genus  Fusarium | HEX | Fus Pb | ACGCCTGGGTYCTTGACAAGC | 5' 6-HEX;3'BHQ1 |
|  |  |  | Fus F | CTCGGWAAGGGTTCYTTC |  |
|  |  |  | Fus R | CCAATGACGGTGACATAGTAG |  |
| **18** | GAPDH | CY5 | QGAPDH-Pb | CAGCGACACCCACTCCTCCACCTT | 5’CY5;3'BHQ2 |
|  |  |  | QGAPDHF | ACTGAGCACCAGGTGGTCT |  |
|  |  |  | QGAPDHR | ATGAGCTTGACAAAGTGGTCG |  |

Table S1. The sequence and probe of the pathogen detectable

| Reaction system | Reagent name | Dosage (μl) | Reaction system | Reagent name | Dosage (μl) |
| --- | --- | --- | --- | --- | --- |
| Reaction system 1/3/5/6 | Reaction solution 1/3/5/6 | 9 | Reaction system 2/4 | Reaction solution 2/4 | 0.4 |
|  | Enzyme mixture 1 | 1 |  | Enzyme mixture 2 | 5 |
|  | ddH_2_O | NA |  | ddH_2_O | 2.1 |
|  | template | 2.5 |  | template | 2.5 |
|  | Total | 12.5 |  | Total | 12.5 |

Table S2. Reagent composition of different systems

| Reaction system | Step | Temperature | Time | Cycles |
| --- | --- | --- | --- | --- |
| Reaction system 1/3/5/6 | Step.1 | 95 | 3min | 1 |
|  | Step.2 | 95 | 10s | 40 |
|  |  | 60 | 30s (fluorescent collection) |  |
|  |  |  |  |  |
| Reaction system 2/4 | Step.1 | 95 | 2min | 1 |
|  | Step.2 | 95 | 5s | 40 |
|  |  | 60 | 15s (fluorescent collection) |  |

Table S3. Detection process of different systems

*Reaction system 1/3/5/6 include pathogens 2, 3, 5, 6, 8, 9, 13, 14, 15, 16, 17, and internal reference

*Reaction system 2/4 include pathogens 1, 4, 7, 10, 11, and 12
